# Iron-Deficiency Anemia Associated With Prostate Cancer, and Ameliorated by Anti-androgen Therapy

**DOI:** 10.7759/cureus.88150

**Published:** 2025-07-17

**Authors:** Tsuneo Takenaka, Choichiro Ozu, Yoko Oshiba, Yutaka Kawakami, Makoto Okuda

**Affiliations:** 1 Nephrology, International University of Health and Welfare, Tokyo, JPN; 2 Urology, Sanno Hospital, Tokyo, JPN; 3 Gynecology, Sanno Hospital, Tokyo, JPN; 4 Hematology, Sanno Hospital, Tokyo, JPN; 5 Surgery, Sanno Hospital, Tokyo, JPN

**Keywords:** anti-androgen, ferritin, hepcidin, malignancy, remote effect

## Abstract

Hepcidin is synthesized in liver and is a master regulator of iron metabolism. Renal anemia was suspected in an 86-year-old man, who was referred to our department. Laboratory tests indicated iron-deficiency anemia (IDA), and ferrous sulphate was initiated. However, no bleeding sites were demonstrated and the response to iron supplementation was not satisfactory. MRI revealed a tumor in the prostate, and prostate biopsy showed poorly differentiated adenocarcinoma that was positive for hepcidin. Anti-androgen therapy ameliorated IDA. Potential roles of hepcidin from prostate cancer (PCa) in the pathogenesis of IDA are discussed in this article.

## Introduction

While iron-deficiency anemia (IDA) prevails in pre-menoposal women, it is less common in post-menoposal women and men [1]. Iron losses occur at a steady rate via turnover of the intestinal epithelium, blood loss, and exfoliation of dead skin cells [2]. Indeed, dietary iron deficiency remains a major health concern with a global prevalence of 16.7% [3]. Hepcidin is a master regulator of iron metabolism [4]. It has been proposed that hepcidin is a mediator of the common clinical syndrome, anemia of chronic diseases. In addition, the overexpression of hepcidin is associated with a hypoferremic, microcytic anemia [5]. Although hepcidin facilitates the sequestration of iron, steady iron loss and hepcidin-induced inhibition of iron absorption may eventually develop iron deficiency [6]. Recent studies reveal that cancer cells express and secrete hepcidin, possibly affecting erythropoiesis [7]. Notably, serum hepcidin level is negatively correlated with hemoglobin (Hb) level in patients with breast cancer [8]. While anemia of chronic diseases is common in various cancers, IDA provides us the clue to detect alimentary tract cancers. Herein, we report a case of IDA associated with prostate cancer (PCa).

## Case presentation

Renal anemia was suspected in an 86-year-old man who was referred to our department. Physical examination revealed anemia, hypertension, and prostate enlargement. Laboratory tests indicated elevated serum creatinine (Scr), low Hb and ferritin levels with small mean corpuscular volume (MCV) (Table 1).

**Table 1:** Clinical course of the patient Serum hepcidin was measured using ELISA assay (R&D Systems) SBP: Systolic blood pressure; DBP: Diastolic blood pressure; Scr: Serum creatinine; Hb: Hemoglobin; MCV: Mean corpuscular volume; PSA: Prostate-specific antigen; F/H: Ratio of log_10_(ferritin) and log_10_(hepcidin)

		Values at first visit	Values at second visit	Values at admission	Values after discharge	Reference range	
Time (month)		0	1	2	4	not applicable	
SBP (mmHg)		168	138	140	136	not applicable	
DBP (mmHg)		86	76	80	78	not applicable	
Scr (mg/dL)		1.84	1.88	1.82	1.85	0.61-1.04	
Hb (g/dl)		7	7.6	7.7	10.9	13.1-16.3	
MCV (fL)		64.8	68.5	67.8	80.8	85-102	
Ferritin (ng/ml)		12	14	14	18	17-291	
Hepcidin (ng/ml)		not analyzed	not analyzed	82	28	not applicable	
PSA (ng/ml)		not analyzed	15.1	not analyzed	0.1	<4.0	
F/H		not analyzed	not analyzed	0.578	0.867	not applicable	

In addition, his urinalysis and C-reactive protein (0.10 mg/dl) were normal. IDA and nephrosclerosis were diagnosed, and ferrous sulphate (105 mg/day) and amlodipine (5 mg/day) were initiated. Surprisingly, fecal occult blood test, esophagogastroduodenoscopic and colonoscopic studies were turned out negative one month later. His blood pressure was controlled without changes in renal function, and amlodipine was continued. His Hb and ferritin levels were only slightly improved (Table 1).

**Figure 1:**
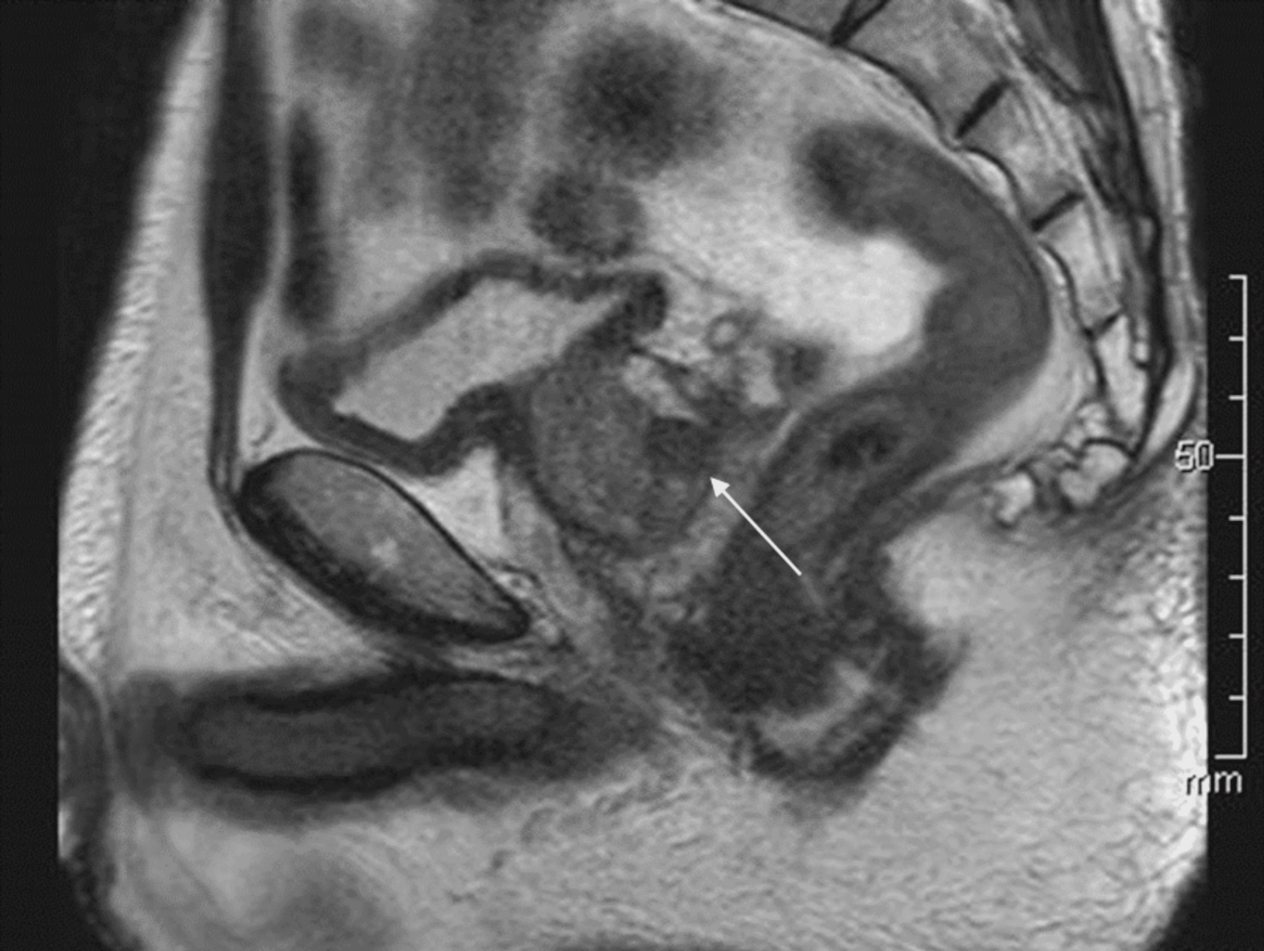
MRI of the prostate T2-weighted image detected a homogeneous low signal mass (10 mm, white arrow) without a capsule.

Thus, IDA seemed to be resistant to oral iron supplementation in this patient, however, iron supplementation was halted because of cramping. His prostate-specific antigen (PSA) level was high (Table 1), and MRI study suggested PCa (Figure 1).

**Figure 2:**
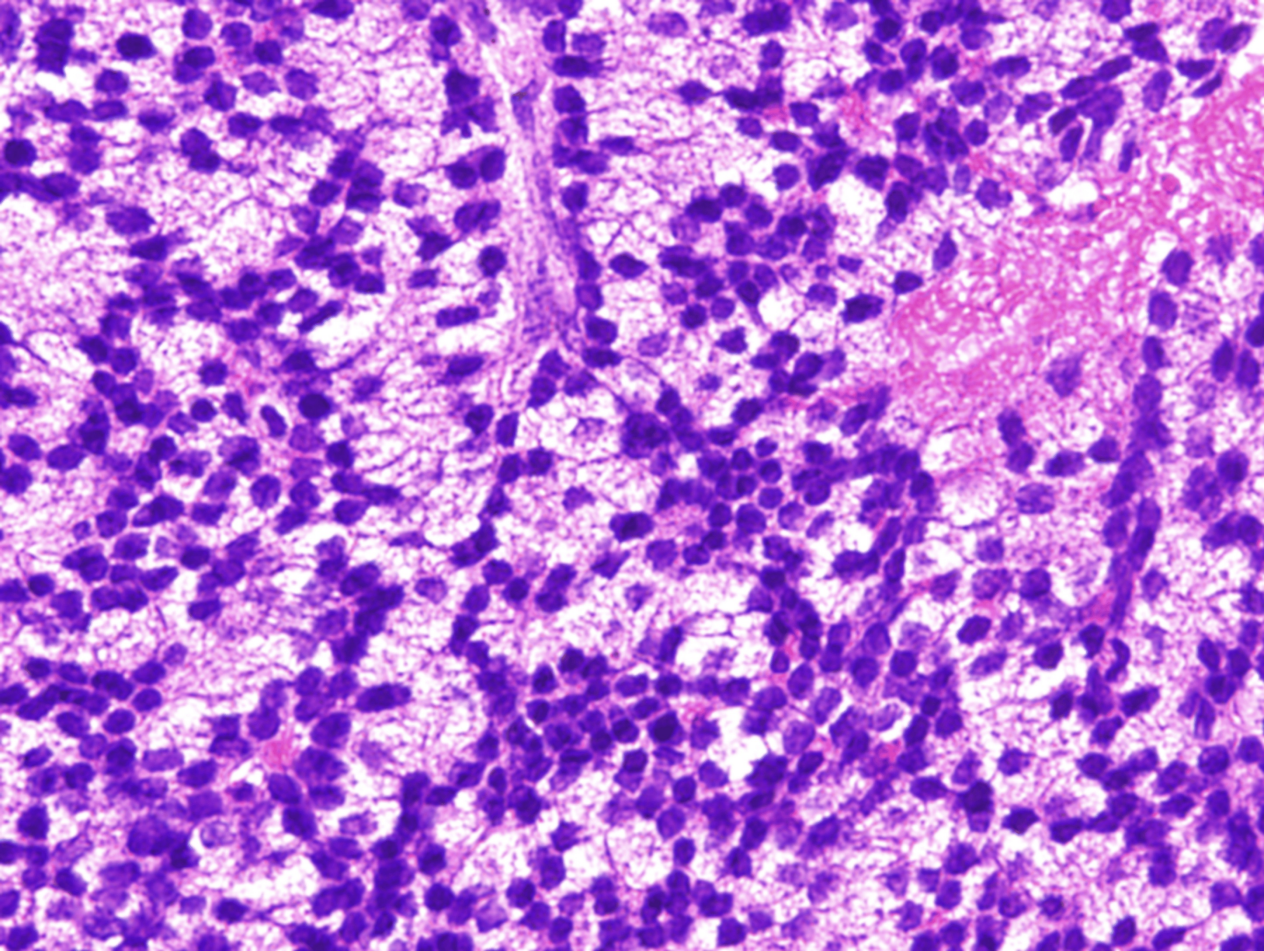
Pathological examination of the prostate tissue sample Numerous atypical adenocarcinoma cells with oval swollen nuclei were observed (Gleason grade group 5), suggesting rapid growth (HE-stain x400). HE: Hematoxylin-eosin

About a month later, he admitted to the hospital for prostate biopsy, which confirmed a poorly-differentiated adenocarcinoma (Figure 2).

**Figure 3:**
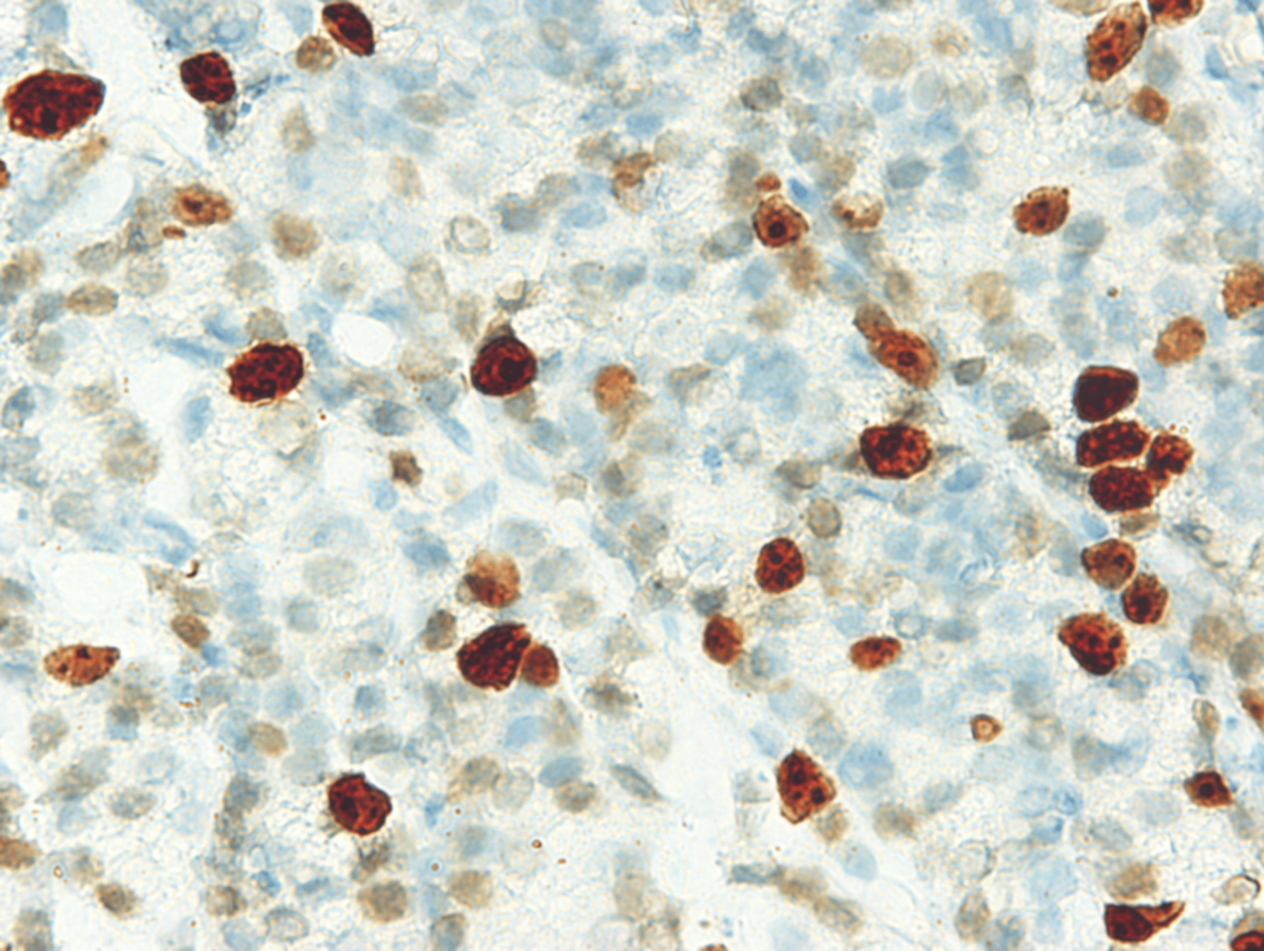
Immunohistochemical analysis of prostate tissue PCa cells were stained positively for hepcidin with varied densities (peroxidase stain x400). An antibody against hepcidin (Santa Cruz Biotechnology) was used. PCa: Prostate cancer

The biopsy was positive for hepcidin (Figure 3) and serum hepcidin level was high (Table 1). He denied surgery and radiotherapy, and chose hormonal therapy with bicalutamide (80 mg/day), an anti-androgen agent. He did not agree to restart iron supplement. Two months later, his Hb, MCV, and ferritin levels increased further, while PSA and hepcidin levels declined (Table 1). Collectively, bicalutamide alone considerably ameliorated IDA.

## Discussion

When iron is sufficient, liver synthesizes hepcidin that triggers ferroportin degradation to inhibit intestinal iron absorption and iron release from macrophages [1]. Under chronic hepcidin excess, steady iron loss could eventually develop iron deficiency [2]. Thus, in physiological conditions, as serum ferritin is increased, hepcidin elevates. Because of various reasons such as reduced iron intake, IDA is a common cause of anemia in older patients. Indeed, the patients with IDA show very low serum hepcidin (sometimes undetectable). In addition, an increased interleukin-6 in the patients with malignancy elevates hepatic synthesis of hepcidin, participating in anemia of chronic diseases [4]. However, C-reactive protein was normal in this patient. The log_10_(ferritin)/log_10_(hepcidin) ratio (F/H) is approximately 0.87 in patients with chronic kidney disease, partly because of hepcidin clearance [9]. However, it was 0.58 in this patient (Table 1). These findings support considerable contribution of extrahepatic production of hepcidin to the elevated serum level in this patient.

Cancer cells are known for their voracious appetites for various nutrients to satisfy their metabolic needs [10]. Similar to other cancers, PCa cells need iron for their survival and growth, including the use of iron for the activity of enzymes that control the expression of androgen receptor, a well-known promotor of PCa. Although ferroportin has not been examined in the case, PCa cells secrete hepcidin to decrease their own ferroportin in autocrine fashion, accumulating iron [10]. Of interest, bicalutamide markedly reduced PSA, suggesting that androgen contributed to the survival and growth of PCa in this patient. The average serum hepcidin level was 42 ng/ml in patients with PCa without metastasis [11]. With declining in PCa differentiation, ferroportin expression of PCa is reduced, possibly accounting for relatively high serum hepcidin level in our patient [12]. Of importance is testosterone, which suppresses hepatic hepcidin release to support erythropoiesis in healthy men [13]. Collectively, the hepcidin positivity of PCa cells and the reversal of IDA by bicalutamide in this patient provide the first clinical evidence that hormonal remote effects of hepcidin from PCa participate in the pathogenesis of IDA. 

## Conclusions

This case suggests that PCa is able to elevate serum hepcidin sufficiently to produce IDA even in the early stage. Because this is the first report of such a case, the accumulation of similar cases is mandatory. However, this report should let us extend the differential diagnosis of IDA to include PCa, especially for older males.
